# FAM53A Affects Breast Cancer Cell Proliferation, Migration, and Invasion in a p53-Dependent Manner

**DOI:** 10.3389/fonc.2019.01244

**Published:** 2019-11-14

**Authors:** Jie Zhang, Mingfang Sun, Miaomiao Hao, Kexin Diao, Jian Wang, Shiping Li, Qixue Cao, Xiaoyi Mi

**Affiliations:** Department of Pathology, College of Basic Medical Sciences, First Affiliated Hospital, China Medical University, Shenyang, China

**Keywords:** breast cancer, cell migration, epithelial-mesenchymal transition (EMT), invasion, metastasis, p53, extracellular-signal-regulated kinase (ERK)

## Abstract

Family with sequence similarity 53-member A (FAM53A) is an uncharacterized protein with a suspected but unclear role in tumorigenesis. In this study, we examined its role in breast cancer. Immunohistochemical staining of specimens from 199 cases of breast cancer demonstrated that FAM53A levels were negatively correlated with p53 status. In the p53 wild-type breast cancer cell line MCF-7, FAM53A overexpression inhibited cell migration, invasion, and proliferation, downregulated the expression of Snail, cyclin D1, RhoA, RhoC, and MMP9, and decreased mitogen-activated protein kinase kinase (MEK) and extracellular-signal regulated kinase (ERK) phosphorylation. Concurrently, it upregulated E-cadherin and p21 expression levels. Interestingly, opposite trends were observed in the p53-null breast cancer cell line MDA-MB-231. The MEK inhibitor PD98059 reduced the biological effects of FAM53A knockdown in MCF-7 cells and FAM53A overexpression in MDA-MB-231 cells, suggesting that FAM53A affects breast cancer through the MEK-ERK pathway. Silencing *TP53* in MCF-7 cells and stably expressing wild-type p53 in MDA-MB-231 cells confirmed that the effects of FAM53A signaling through the MEK/ERK pathway depended on the p53 status of the cells. These results suggest that FAM53A acts as a tumor suppressor in p53-positive breast cancer by modulating the MEK-ERK pathway, but may be a potential candidate for targeted anticancer therapies in p53-negative breast cancer.

## Introduction

The mitogen-activated protein kinase kinase (MEK)-extracellular-signal regulated kinase (ERK) pathway is an evolutionarily conserved signal transduction pathway of crucial importance in tumorigenesis. It is often aberrantly activated in malignant tumors, resulting in signal amplification during tumor invasion and metastasis (1, 2). Abnormal activation leads to loss of differentiation and apoptosis and increased proliferation and invasion, causing tumorigenesis and eventual metastasis (3–6). The MEK-ERK pathway is activated at the cell membrane by Ras, which activates Raf, starting a phosphorylation cascade that results in the sequential activation of MEK1/2 and ERK1/2 (7–12). ERK expression is significantly higher in breast cancer tissue than in benign hyperplastic breast tissue, and ERK phosphorylation is increased in severe atypical hyperplasia and breast cancer tissues compared with that in benign proliferating breast tissue, suggesting that abnormally increased ERK activation plays an important role in the development of atypical hyperplasia into cancer, and can stimulate the proliferation of breast cancer cells (13). Phosphorylated ERK (p-ERK) enters the nucleus to phosphorylate specific transcription factors such as c-Myc and c-Jun (14). Decreased p-ERK reduces the expression of matrix metalloprotease (MMP)1 and MMP9, significantly inhibiting breast cancer invasiveness (14, 15). In breast cancer cells, ERK inactivation is accompanied by the inactivation of cyclin D1 and BCL2, leading to apoptosis (15).

Family sequence similarity (FAM) genes are families of uncharacterized genes of similar protein sequence. Several of these families have been linked to the development of tumors, including breast cancer, non-small cell lung cancer, lung adenocarcinoma, renal cell carcinoma, prostate cancer, colorectal cancer, and esophageal squamous cell carcinoma, where they are thought to play important roles in proliferation, apoptosis, migration, and invasion (16–23). FAM53 is a vertebrate-specific family of proteins that bind to transcriptional regulators of proliferation and neural tube development, encoded by three homologous genes: *FAM53A, FAM53B*, and *FAM53C* (24–27). FAM53A, also known as dorsal neural tube nuclear protein, is thought to play an important role in neurodevelopment by specifying the fate of dorsal cells within the neural tube (28, 29). Expression quantitative trait loci variants of *FAM53A* identified in *TP53*-based interaction analysis are associated with the use of therapeutic doxorubicin in breast cancer (27). In the triple-negative *TP53*-missense mutant breast cancer cell line MDA-MB-231, downregulation of FAM53A increased doxorubicin resistance. However, in the luminal B p53-truncated mutant line MDA-MB-361 and the luminal A p53-wild-type line MCF7, downregulation of FAM53A resulted in increased sensitivity to doxorubicin (27). The role of FAM53A in breast cancer is unclear, and its relevance to the clinical pathology of breast cancer has not been reported. In this study, we examined the expression and localization of FAM53A in breast cancer tissues and cell lines. We then altered FAM53A expression in two breast cancer cell lines to explore its effects on the cells and gain mechanistic insight into how FAM53A affects cancer.

## Experimental procedures

### Patients and Specimens

Primary tumor specimens were obtained from 199 patients diagnosed with invasive ductal carcinoma who underwent complete resection in the First Affiliated Hospital of China Medical University between 2011 and 2013. Patients whose tissue samples were used in this research provided written informed consent. This study was approved by the local institutional review board of China Medical University.

### Cell Culture and Treatment

MCF-10A, MCF-7, T47D, MDA-MB-231, BT-474, and BT-549 cell lines were obtained from the Shanghai Cell Bank of the Chinese Academy of Sciences (Shanghai, China) and identified by short tandem repeat (STR) DNA analysis. The cells were cultured and frozen, and experiments were performed after 10 passages. MCF-10A cells were cultured in 1:1 Dulbecco's modified Eagle's medium (DMEM)/F12 (Gibco, Waltham, MA, USA) supplemented with 5% horse serum, 10 μg/mL insulin (Sigma-Aldrich Co, St. Louis, MO, USA), and 20 ng/mL epidermal growth factor (EGF). MDA-MB-231 cells were cultured in L15 (Gibco) supplemented with 10% fetal bovine serum (FBS). MCF7 and T47D cell lines were cultured in DMEM (Gibco) supplemented with 10% FBS. BT-474 and BT549 cells were cultured in Roswell Park Memorial Institute (RPMI)-1640 (Gibco) supplemented with 10% FBS. Cells were cultured in sterile culture flasks in a 5% CO_2_ incubator at 37°C and subcultured every 2 days by trypsinization.

### Immunohistochemistry

Surgically removed tumor specimens were fixed in 10% neutral formalin, embedded in paraffin, and continuously cut into 4-μm thick sections. The tissue slices were baked in an oven at 70°C for 2 h; then the sections were dewaxed in xylene, absolute ethanol, gradient alcohol, and distilled water, and boiled in 0.01 M citrate buffer (pH 6.0) at high temperature and high pressure for 2 min. Endogenous peroxidase activity was blocked with 0.3% hydrogen peroxide and sections were incubated with 5% normal goat serum for 30 min at 20°C to reduce non-specific binding. Tissue sections were then incubated with FAM53A antibody (1:100 dilution; see Table S1 for information on antibodies used in the study) overnight at 4°C. The reaction was observed using an Elivision super HRP (mouse/rabbit) immunohistochemistry kit (Maixin-Bio, Shenzhen, China) and 3,3′-diaminobenzidine (DAB). The nuclei were counterstained with hematoxylin.

FAM53A expression levels were evaluated based on the percentage of positive cells (PP) and the staining intensity (SI) within the whole tissue section. FAM53A staining intensity was evaluated semi-quantitatively using the immune response score (IRS) and calculated as follows: IRS = PP × SI; where PP: 0, no dye; 1, 1–25%; 2, 26–50%; 3, 51–75%; and 4, 76–100%; and SI: 0, no staining; 1, weak staining; 2, medium staining; and 3, strong staining. The scores for each tumor sample were multiplied to give a final score of 0–9; tumor samples with scores >3 were classified as having high FAM53A expression, while samples with scores ≤3 were classified as having low FAM53A expression.

### Plasmid Transfection, siRNA Interference, and Inhibitor Treatments

Transfection was performed using Xfect Transfection Reagent (Takara Bio USA, Mountain View, CA, USA) according to the manufacturer's instructions. The plasmids pCMV6-ddk-myc and pCMV6-ddk-myc-FAM53A were purchased from Origene (Rockville, MD, USA). One day before transfection, cells were plated in 2 mL of complete growth medium, aiming for 50–70% confluency at the time of transfection. Plasmid DNA (5 μg) was added to Xfect Reaction Buffer to a final volume of 100, and 1.5 μL Xfect Polymer was added. After a 10 min incubation at room temperature (15–25°C), the solution was added dropwise to the cell culture medium. Cells were incubated at 37°C for 4 h, then the medium was replaced with 2 mL of fresh complete growth medium and the cells were incubated at 37°C for 48 h. FAM53A (sc-88998) and non-targeted control (NC; sc-37007) siRNAs were purchased from Santa Cruz Biotechnology (Dallas, TX, USA), and transfected into cells using HiPerFect Transfection Reagent (Qiagen, Hilden, Germany) according to the manufacturers' protocols. Cells were plated in 2 mL of complete growth medium, aiming for 30–50% confluence at the time of siRNA transfection. The next day, 37.5 ng of siRNA was added to 100 μL of serum-free culture medium, 3 μL of HiPerFect Transfection Reagent was added, and the solution was mixed by vortexing and incubated for 5–10 min at room temperature to allow the formation of transfection complexes. The complexes were then added to the cells, which were incubated for 48 h prior to analysis.

PD98059, a MEK inhibitor, was purchased from Selleck Chemicals (Houston, TX, USA). A stock solution was generated by dissolving PD98059 in dimethyl sulfoxide (DMSO). This solution was added to MCF-7 and MDA-MB-231 cells at final concentrations of 10 μM for 1 h and 25 μM for 2 h, respectively, prior to analysis.

### Immunofluorescence

Breast cancer cells cultured in 24-well plates for 24 h were fixed in 2% paraformaldehyde for 15 min, blocked in 5% BSA for 2 h, and incubated with anti-FAM53A antibody (1:100) at 4°C overnight. Cells were then incubated with a tetramethylrhodamine-labeled secondary antibody at room temperature for 2 h in the dark; the nuclei were counterstained with 4′,6-diamidino-2-phenylindole. Images were captured using an Olympus FV1000 laser scanning confocal microscope (Olympus, Tokyo, Japan).

### Western Blot Analysis

Cells and tumor tissues were lysed in lysis buffer (Thermo Fisher Scientific), and total protein was quantified using the Bradford method. Proteins (80 μg/lane) were separated by 10% SDS-PAGE and then transferred to polyvinylidene difluoride membranes (Millipore, Billerica, MA, USA). Membranes were incubated overnight at 4°C with the appropriate primary antibody (Table S1) and for 2 h at room temperature with horseradish peroxidase-labeled secondary antibody, then visualized with ECL (Thermo Fisher Scientific, Waltham, MA, USA) on a BioImaging System (UVP Inc., Upland, CA, USA). Protein levels were analyzed using the ImageJ software (National Institutes of Health, Bethesda, MD, USA), with glyceraldehyde 3-phosphate dehydrogenase (GAPDH) as a loading control.

### Cell Proliferation and Colony Formation Assays

Cell viability was measured by the mitochondrial reduction of 3-[4,5-dimethylthiazole-2-yl]-2,5-diphenyltetrazolium bromide (MTT). Cells (3,000/well) were seeded in 96-well plates in medium containing 10% FBS and have used a separate blank (100 ml of fresh medium). MTT solution (10 μL/well) was added and samples were incubated for 4 h. Then, the medium was aspirated from each well, and the obtained MTT formazan was dissolved in 150 μL DMSO. Finally, the absorbance was measured at 490 nm using a microplate reader daily, and the OD value of the blank was subtracted from the absorbance at a given time. We calculated the relative ratio and plotted the cell curve.

For colony formation experiments, cells (1,000/dish) were seeded in 40-mm dishes and incubated for 10–15 days in medium containing 10% fetal calf serum, which was changed every 3 days. After incubation, the cells were washed with PBS and stained with hematoxylin, and the number of colonies with >50 cells were counted. At least three independent experimental replicates were performed.

### Cell Migration and Invasion Assays

Cell migration and invasion experiments were performed using 24-well Transwell chambers (Costar, Cambridge, MA, USA) with a pore size of 8 μm. For the invasion assay, the upper chamber of the Transwell chamber was coated with 100 μL Matrigel (1:9 dilution; BD Biosciences); for cell migration assays, no Matrigel was added. Approximately 24 h after transfection, the cells were trypsinized, and 1 × 10^5^ cells in 100 μL of medium supplemented with 2% FBS were transferred to the upper chamber. Medium supplemented with 10% FBS was added to the lower chamber as a chemoattractant. After 18 h of incubation, the chamber was removed and stained with hematoxylin, and cells that migrated through the chamber membrane were counted using an Olympus FV1000 laser-scanning confocal microscope (Olympus, Tokyo, Japan). At least three independent experimental replicates were performed.

### Statistical Analysis

Correlations between FAM53A expression and clinicopathological features of breast cancer were analyzed using the chi-squared test. For experiments involving cells, differences between the control and experimental groups were compared by paired *t*-test. *P* < 0.05 was considered statistically significant.

## Results

### FAM53A Expression in Breast Cancer Cells Is Associated With p53

**Figure 1:**
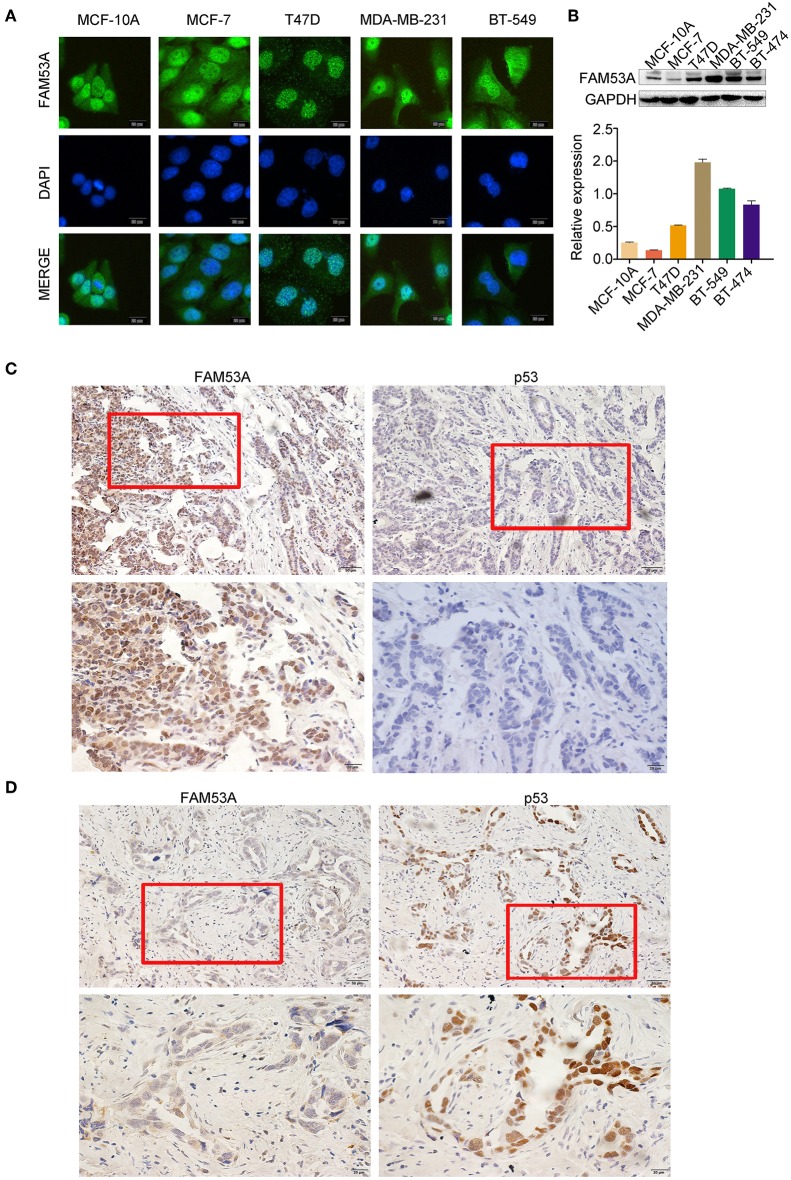
FAM53A expression in breast cancer cell lines and tissues. **(A)** FAM53A expression in breast cancer cell lines was analyzed by immunofluorescence. **(B)** FAM53A protein levels in five breast cancer cell lines and a normal human mammary epithelial cell line (MCF-10A) were assessed by western blotting. **(C,D)** The relationship between FAM53A expression and p53 levels in breast cancer tissues was analyzed by immunohistochemistry.

We examined the localization of FAM53A in the breast cancer cell lines MCF-7, T47D, MDA-MB-231, and BT-549 and the non-malignant human mammary epithelial cell line MCF-10A by immunofluorescence and observed its presence in the cytoplasm and nucleus (Figure 1A). FAM53A was expressed at significantly lower levels in the p53-wild-type breast cancer cell line MCF-7 compared with the normal human mammary epithelial cell line MCF-10A, whereas in the p53-mutant breast cancer cell lines T47D, MDA-MB-231, BT-549, and BT-474, FAM53A was highly expressed, particularly in MDA-MB-231 cells (Figure 1B). We therefore selected MCF-7 and MDA-MB-231 cells for subsequent experiments. To investigate the association between FAM53A expression and clinicopathological features of breast cancer, we selected 199 breast cancer tissues for immunohistochemical staining. As shown in Table 1, FAM53A levels were negatively correlated with wild-type p53 (*P* < 0.001; Figures 1C,D), but had no significant correlation with age (*P* = 0.781); tumor size (*P* = 0.110); TNM stage (*P* = 0.056); lymph node metastasis (*P* = 0.996); or estrogen receptor, progesterone receptor, or human epidermal growth factor receptor 2 (HER-2) status (*P* = 0.069). Therefore, we next examined the effects of modulating FAM53A levels in the presence and absence of functional p53.

**Table 1:** Correlation between FAM53A expression and clinicopathological characteristics of invasive breast cancer.

**Clinicopathological feature**	***N***	**FAM53A**		***P*-value**
		**Positive**	**Negative**	
All cases	199	70	129	
**Age (years)**				
≤50	105	36	69	0.781
>50	94	34	60	
**Tumor size**				
≤5.0 cm	92	27	65	0.110
>5.0 cm	107	43	64	
**TNM stage**				
I/II	109	32	77	0.056
III	90	38	52	
**Lymph node metastasis**				
Negative	119	42	77	0.996
Positive	80	28	52	
**p53 status**				
Negative	115	53	62	<0.001
Positive	84	17	67	
**ER, PR, and HER-2 Status**				
Non-TNBC	176	58	118	0.069
TNBC	23	12	11	

*TNBC, triple-negative breast cancer; ER, estrogen receptor; PR, progesterone receptor*.

### FAM53A Inhibits Proliferation, Migration, and Invasion in the p53-Wild-Type Breast Cancer Cell Line MCF-7

**Figure 2:**
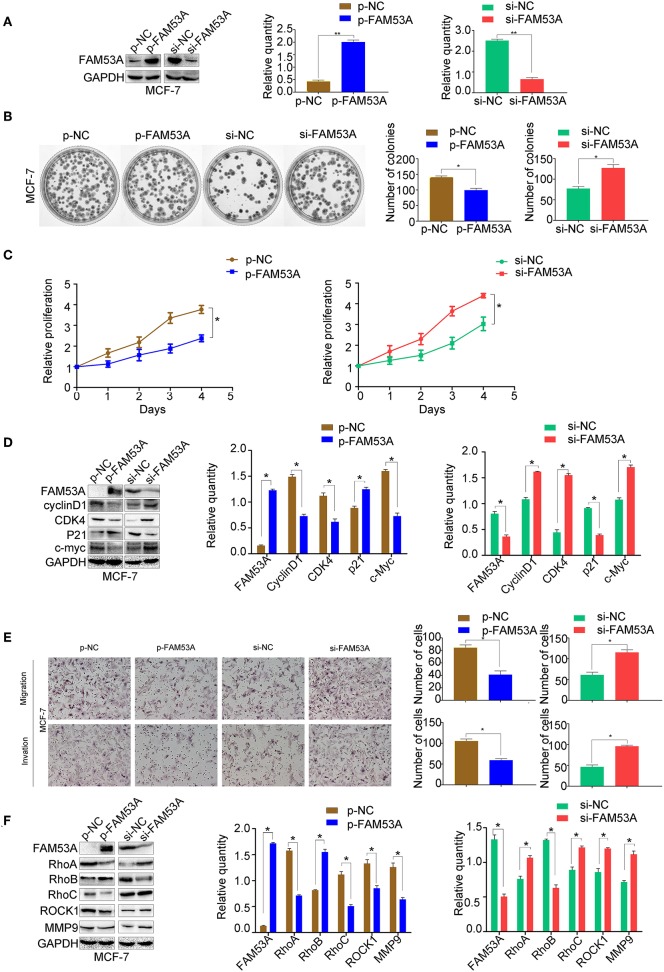
FAM53A inhibits proliferation, migration, and invasion in p53-wild-type breast cancer cells. **(A)** MCF-7 cells were transfected with FAM53A-specific (si-FAM53A) or control (NC) siRNA, or with a FAM53A expression (p-FAM53A) or vehicle (p-NC) plasmid. Western blotting was performed to evaluate transfection and silencing efficiency. **(B)** Representative images and quantification of colony formation assays. **(C)** MTT assay results. **(D)** Expression changes in proliferation-related proteins. **(E)** Cell invasion and migration assays. **(F)** Expression of proteins associated with cell migration and invasion. For all panels, *P < 0.05; **P < 0.01.

To examine the effects of FAM53A expression on tumorigenesis-related processes in a wild-type p53 cell line, FAM53A was both overexpressed and depleted in MCF-7 cells (Figure 2A). When FAM53A was overexpressed, colony formation ability and proliferation were significantly decreased; accordingly, FAM53A depletion resulted in increased colony formation ability and proliferation (Figures 2B,C). We also performed western blot analysis to measure the levels of several proteins related to proliferation. When FAM53A was overexpressed, cyclin D1, cyclin-dependent kinase 4 (CDK4), and c-Myc expression was downregulated, while p21 expression was increased; FAM53A depletion resulted in the opposite effects on these proteins (Figure 2D). FAM53A overexpression inhibited the migration and invasion of MCF-7 cells, while FAM53A depletion promoted these processes (Figure 2E). Changes in FAM53A protein levels also affected the expression of proteins involved in cell migration and invasion, with decreased RhoA, RhoC, Rho kinase 1 (ROCK1), and MMP9 expression and increased RhoB expression after FAM53A overexpression. Expression of RhoA, RhoC, ROCK1, and MMP9 correspondingly increased after FAM53A depletion, along with decreased RhoB expression (Figure 2F). These results suggest that FAM53A inhibits proliferation, migration, and invasion in the presence of wild-type p53.

### FAM53A Promotes Proliferation, Migration, and Invasion in the p53-Mutant Breast Cancer Cell Line MDA-MB-231

**Figure 3:**
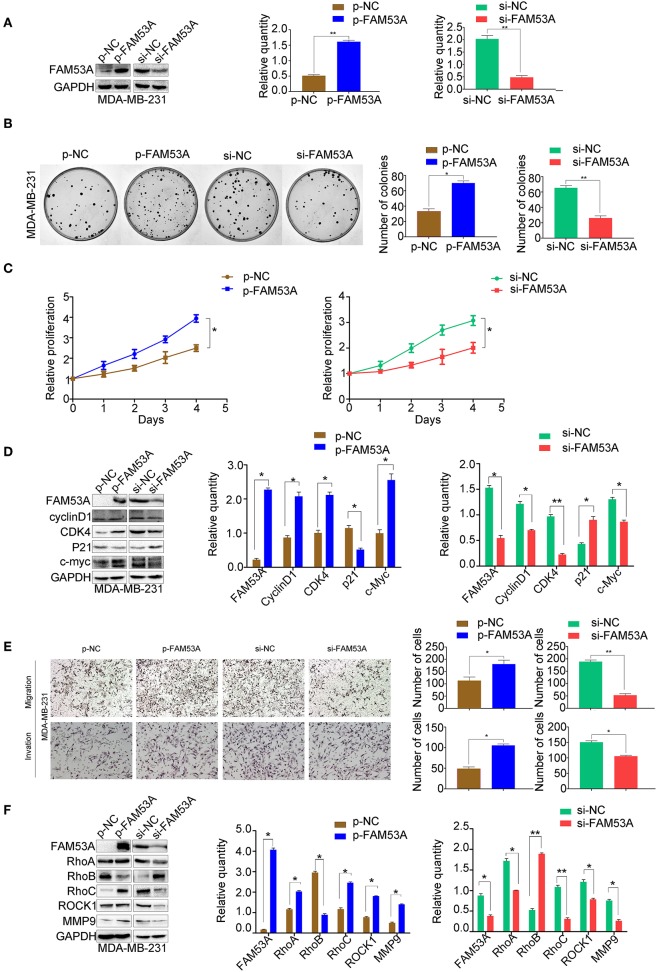
FAM53A promotes proliferation, migration, and invasion in p53-mutant breast cancer cells. **(A)** MDA-MB-231 cells were transfected with FAM53A-specific (si-FAM53A) or control (NC) siRNA, or with a FAM53A expression (p-FAM53A) or vehicle (p-NC) plasmid. Western blotting was performed to evaluate transfection and silencing efficiency. **(B)** Representative images and quantification of colony formation assays. **(C)** MTT assay results. **(D)** Expression changes in proliferation-related proteins. **(E)** Cell invasion and migration assays. **(F)** Expression of proteins associated with cell migration and invasion. For all panels, *P < 0.05; **P < 0.01.

FAM53A overexpression and depletion was also performed in MDA-MB-231 cells (Figure 3A). In this cell line, overexpression of FAM53A increased colony formation ability and proliferation, while depletion had the opposite effects (Figures 3B,C). The expression of proteins important for proliferation showed opposite trends with FAM53A modulation to those observed in MCF-7 cells; namely, FAM53A overexpression increased cyclin D1, CDK4, and c-Myc levels, while decreasing p21 levels. The depletion of FAM53A expression resulted in decreased cyclin D1, CDK4, and c-Myc and increased p21 (Figure 3D). FAM53A overexpression promoted migration and invasion in MDA-MB-231 cells, while depletion inhibited these processes (Figure 3E). FAM53A overexpression increased RhoA, RhoC, ROCK1, and MMP9 expression and decreased RhoB expression, and the opposite trends were observed with FAM53A depletion (Figure 3F). Taken together, the above results indicate that FAM53A has opposing effects on proliferation, migration, and invasion of breast cancer cell lines with wild-type and mutated p53.

### FAM53A Differentially Affects the Expression of Epithelial-Mesenchymal Transition (EMT)-Related Proteins

**Figure 4:**
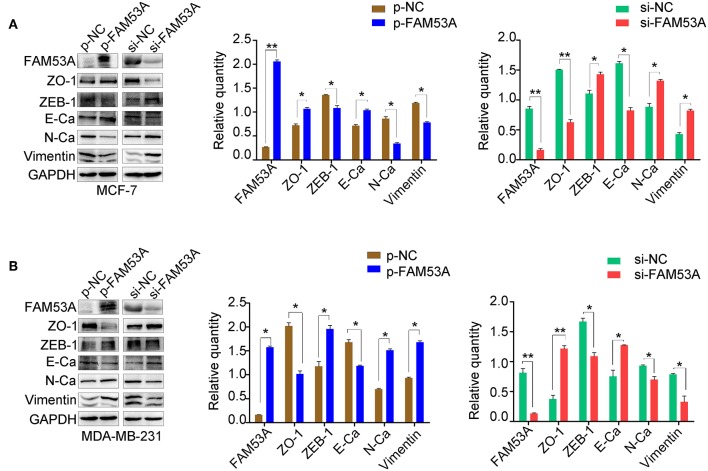
FAM53A regulates the levels of EMT-related proteins. **(A,B)** Expression changes in EMT-related proteins in MCF-7 and MDA-MB-231 cells, respectively. E-Ca, E cadherin; N-Ca, N cadherin. *P < 0.05; **P < 0.01.

EMT has dramatic effects on tumor cell behavior, as it results in changes in cell polarity and the formation of tight junctions between cells, a gradual loss of adhesion leading to cell invasiveness and migration, and the expression of a large number of extracellular matrix components that affect proliferation, migration, and invasion. As the above experiments demonstrated that FAM53A affects the proliferation, migration, and invasion of breast cancer cell lines, we next explored whether FAM53A regulation is a potential inhibitory mechanism against EMT by examining the expression of EMT-related proteins after modulating FAM53A levels. FAM53A overexpression in MCF-7 cells increased ZO-1 and E-cadherin expression and decreased zinc finger E-box binding homeobox 1 (ZEB1), N-cadherin, and vimentin expression; depletion of FAM53A had the opposite effects on these proteins (Figure 4A). We also examined the expression of EMT-related proteins in MDA-MB-231 cells. FAM53A overexpression decreased the expression of ZO-1 and E-cadherin and increased the expression of ZEB1, N-cadherin, and vimentin, while FAM53A depletion had the opposite effects (Figure 4B).

### FAM53A Regulates Breast Cancer Cells Through the MEK/ERK Signaling Pathway

**Figure 5:**
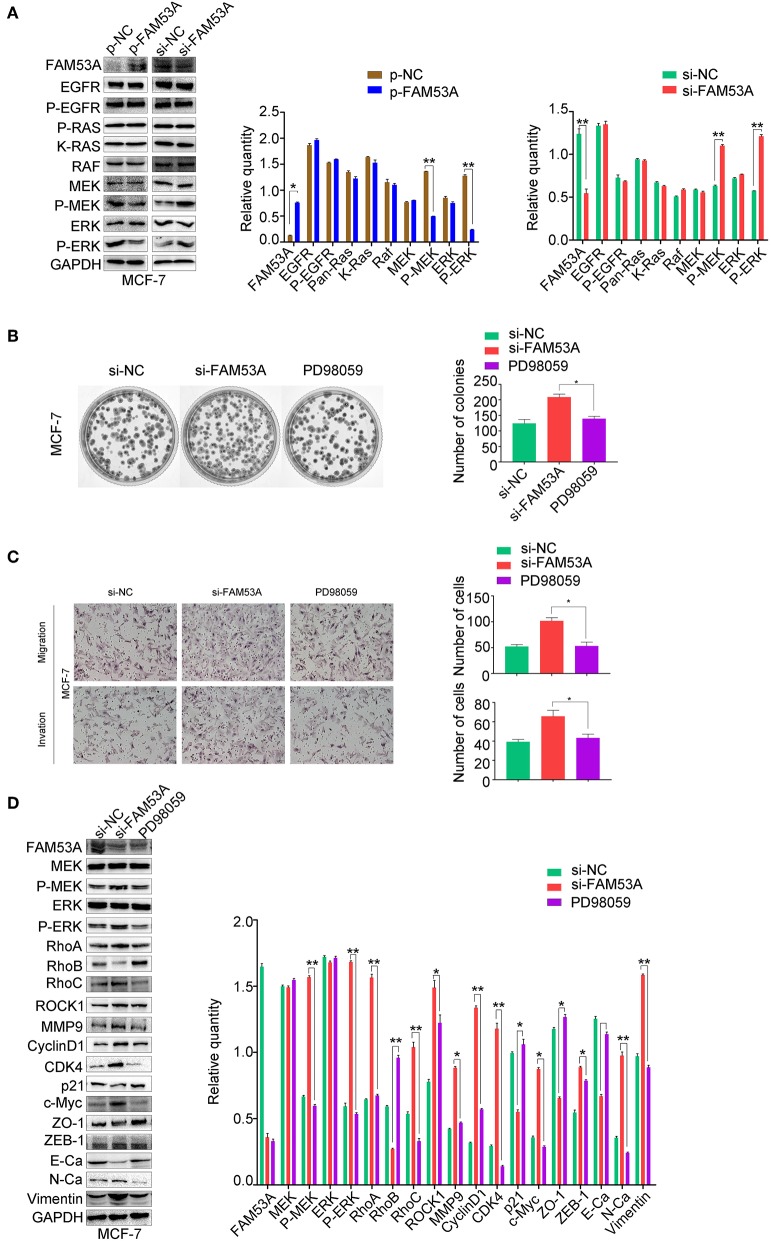
FAM53A regulates MCF-7 cells via the MEK/ERK signaling pathway. MCF-7 cells transfected with control or FAM53A siRNA were treated with or without PD98059 and analyzed for **(A)** changes in the expression and activation of MEK/ERK signaling proteins, **(B)** colony formation ability, **(C)** migration and invasion, and **(D)** expression of proteins involved in cell proliferation, migration, and invasion. For all panels, *P < 0.05; **P < 0.01. E-Ca, E cadherin; N-Ca, N cadherin.

We next sought to gain mechanistic insight into the effects of FAM53A on breast cancer cells. FAM53B, a homolog of FAM53A, is involved in the regulation of Wnt/β-catenin signaling and β-catenin nuclear localization (26). However, a Wnt/β-catenin signaling pathway-related protein assay found no significant changes upon modulation of FAM53A levels (Figure S1). The MEK/ERK signaling pathway is crucial for the proper regulation of proliferation, migration, and invasion, so we next examined the effects of FAM53A on MEK/ERK activation. In MCF-7 cells, FAM53A overexpression resulted in decreased p-MEK1/2 and p-ERK1/2, indicating decreased pathway activation. Conversely, FAM53A depletion significantly increased the phosphorylation of MEK1/2 and ERK1/2 (Figure 5A). Modulation of FAM53A levels had no effect on EGF receptor (EGFR), Ras, Raf, MEK1/2, or ERK1/2 levels, nor did it affect EGFR phosphorylation.

To confirm that FAM53A functions through the MEK/ERK signaling pathway, we employed the specific MEK/ERK pathway inhibitor PD98059. While FAM53A depletion significantly activated MEK/ERK signaling, PD98059 blocked this activation. PD98059 treatment also blocked the increased colony formation activity observed after FAM53A depletion, and rescued the increased migration and invasion, confirming that FAM53A regulates MCF-7 cells through the MEK/ERK signaling pathway (Figures 5B,C). We also examined the expression of proliferation- and EMT-related proteins with PD98059 treatment and found that MEK/ERK inhibition restored protein levels to those observed without FAM53A depletion (Figure 5D). These experiments demonstrate that FAM53A inhibits proliferation, migration, and invasion and negatively regulates the EMT in p53 wild-type breast cancer cells through the MEK/ERK pathway.

**Figure 6:**
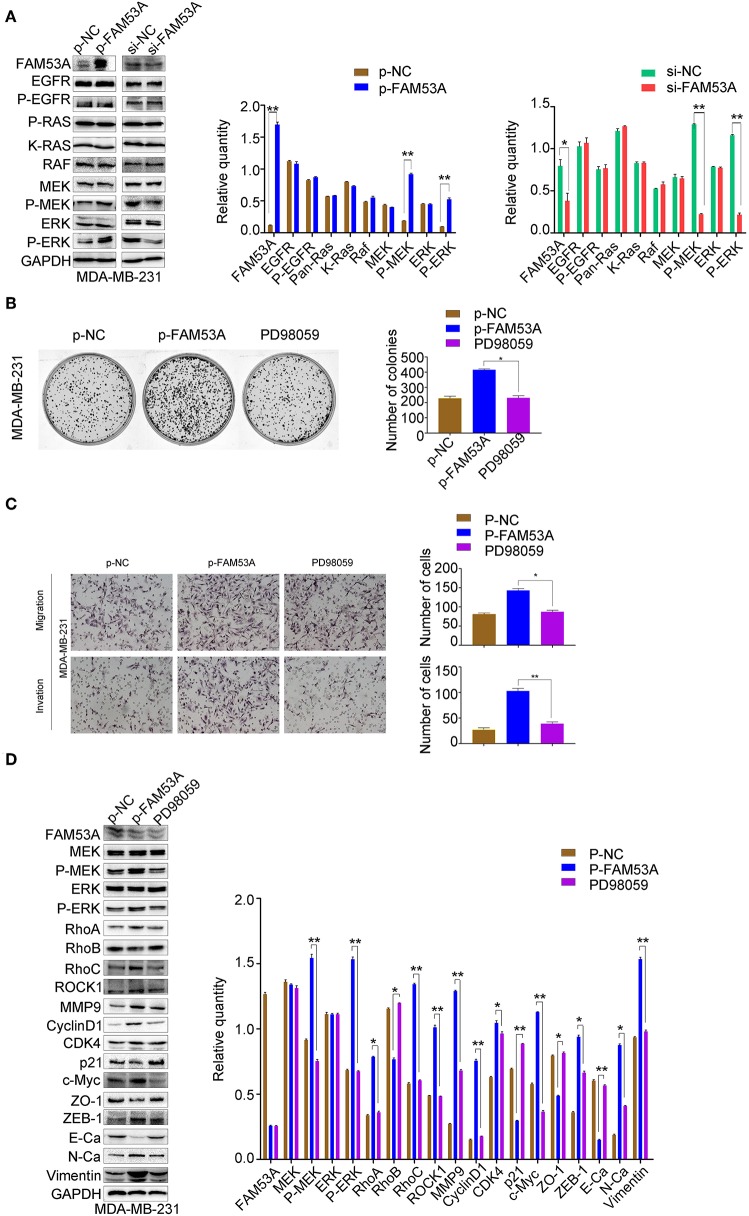
FAM53A regulates MDA-MB-231 cells via the MEK/ERK signaling pathway. MDA-MB-231 cells were transfected with control or FAM53A overexpression plasmids, treated with or without PD98059, and analyzed for **(A)** changes in the expression and activation of MEK/ERK signaling proteins, **(B)** colony formation ability, **(C)** migration and invasion, and **(D)** expression of proteins involved in cell proliferation, migration, and invasion. For all panels, *P < 0.05; **P < 0.01. E-Ca, E cadherin; N-Ca, N cadherin.

In MDA-MB-231 cells, MEK1/2 and ERK1/2 phosphorylation increased with FAM53A overexpression and decreased with its depletion, while EGFR, Ras, Raf, MEK, and ERK levels did not significantly change (Figure 6A). PD98059 rescued the increased colony formation caused by FAM53A overexpression, as well as the increased migration and invasion, confirming that the effects of FAM53A are mediated through MEK/ERK signaling in this cell line as well (Figures 6B,C). PD98059 also reversed the effects of FAM53A overexpression on the expression of proliferation- and EMT-related proteins (Figure 6D).

### The Opposing Effects of FAM53A on MCF-7 and MDA-MB-231 Cells Depend on Their p53 Status

**Figure 7:**
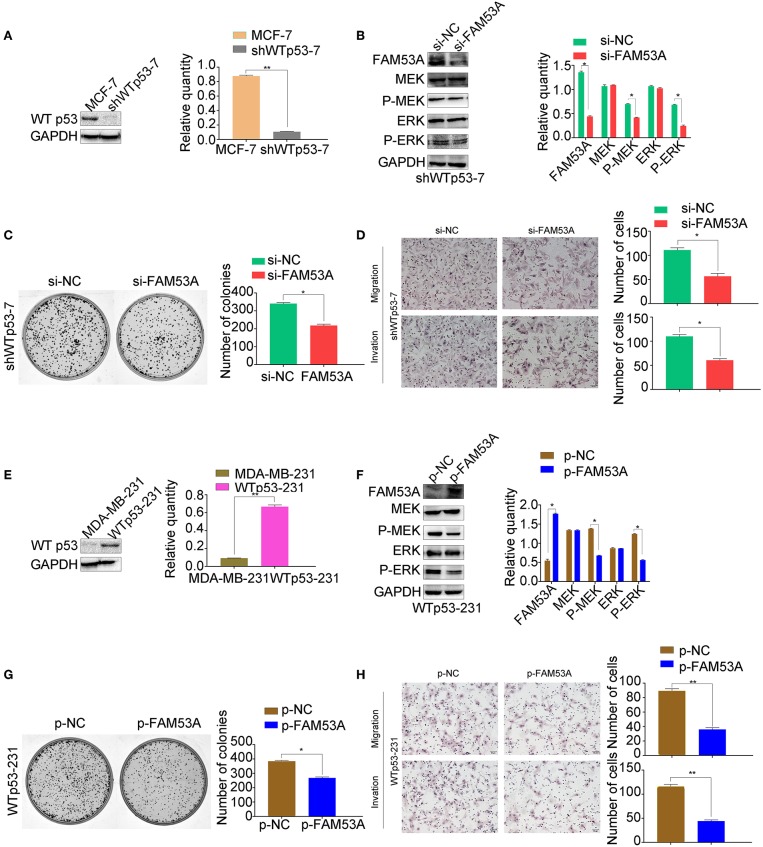
The opposing effects of FAM53A on MCF-7 and MDA-MB-231 cells depend on their p53 status. **(A)** An MCF-7 cell line with stable depletion of wild-type p53 (shWTp53-7) was constructed and evaluated for **(B)** changes in the expression and activation of MEK/ERK signaling proteins, **(C)** colony formation ability, and **(D)** migration and invasion. **(E)** An MDA-MB-231 cell line stably expressing wild-type p53 (WTp53-231) was constructed, transfected with control or FAM53A overexpression plasmids, and evaluated for **(F)** changes in the expression and activation of MEK/ERK signaling proteins, **(G)** colony formation ability, and **(H)** migration and invasion. For all panels, *P < 0.05; **P < 0.01.

Interestingly, our results indicated that FAM53A has opposite effects on the MCF-7 and MDA-MB-231 cell lines. As the only positive correlation between FAM53A expression and the clinicopathological characteristics of invasive breast cancer was p53 status (Table 1), we next investigated whether the differential effects of FAM53A in these lines was due to the presence or absence of functional p53. An MCF-7 cell line with stable expression of a p53 shRNA was generated, which displayed decreased FAM53A expression compared with the parental line (Figure 7A). Depletion of p53 reversed the trends observed upon FAM53A depletion in the parental line, decreasing MEK/ERK activation (Figure 7B), colony formation (Figure 7C), and migration and invasion (Figure 7D).

Next, we constructed an MDA-MB-231 cell line stably expressing wild-type p53 (Figure 7E) and subjected it to the same analysis. FAM53A overexpression in WTp53-231 cells inhibited MEK/ERK activation (Figure 7F), colony formation (Figure 7G), and migration and invasion (Figure 7H).

## Discussion

While FAM53A has yet to be directly linked to tumorigenesis, studies have shown that members of the FAM53 protein family bind to transcriptional regulators that regulate cell proliferation, suggesting potential effects on cancer development. To the best of our knowledge, this is the first demonstration of the relevance of FAM53A expression in cancer. The results indicate that FAM53A levels are negatively correlated with wild-type p53, suggesting a link between the role of FAM53A in breast cancer and p53 status.

FAM53A was shown to affect the sensitivity of breast cancer cell lines to doxorubicin, with opposing effects on breast cancer cell lines with different p53 status (27). However, the effects of FAM53A on breast cancer cells and their related mechanisms were unknown. Our study demonstrates that overexpression of FAM53A reduces proliferation, migration, and invasion ability in the p53-wild-type breast cancer cell line MCF-7, but promoted these abilities in the p53-mutant line MDA-MB-231. We had also selected the p53-wild-type lung cancer cell line A549 and the p53-mutant breast cancer cell line SK-BR3, the results are consistent with the previous conclusions (Figure S2). Mechanistically, the results indicate that FAM53A affects cell proliferation by regulating the p21-cyclin D1-CDK4 signaling axis. In addition, FAM53A may affect the migration and invasion of breast cancer cells by regulating the expression of RhoA, RhoB, RhoC, ROCK1, and MMP9. Through these proteins, FAM53A could drastically affect the behavior of malignant cells.

EMT occurs during tumor invasion and metastasis and represents an important milestone in tumor progression. EMT is mainly characterized by loss of epithelial cell polarity, loss of adhesion between cells, and acquisition of mesenchymal cell characteristics, accompanied by enhanced migration ability (30, 31). Our results indicate that FAM53A affects several key proteins in EMT. Moreover, these effects were consistently opposite between the p53-positive and -negative cells.

Our result demonstrated that FAM53A inhibits MEK and ERK activity in p53 wild-type breast cancer cells, but activates these enzymes in p53-mutant cells. The use of PD98059 to antagonize ERK activation after FAM53A overexpression or depletion indicated that FAM53A affects breast cancer proliferation, migration, and invasion via the MEK/ERK signaling pathway. Further mapping of this pathway will allow more precise determination of the mechanism of FAM53A action.

As a key tumor suppressor gene involved in the regulation of the cell cycle, apoptosis, senescence, and DNA repair, somatic *TP53* mutations are estimated to occur in 20–30% of cancer cases, including breast cancer (31, 32). The prognosis of breast cancer is closely related to lymph node metastasis, which *TP53* mutations can be used clinically to predict (32). Importantly for breast cancer, *TP53* status may be associated with estrogen receptor (ER), HER-2, and Ki-67 status, with important synergistic or regulatory effects (33, 34). When *TP53* is mutated, normal regulation of these biomarkers is lost, promoting lymph node metastasis. The effects of FAM53A levels on the sensitivity of breast cancer to doxorubicin is correlated with p53 status (27), and our previous immunohistochemical staining demonstrated that FAM53A colocalizes with p53. In this study, FAM53A inhibited proliferation, migration, and invasion by inhibiting MEK/ERK signaling in p53 wild-type cells, while in p53 negative breast cancer cells, FAM53A activated MEK/ERK signaling, promoting these behaviors. This suggests that the role of FAM53A is reversed with loss of p53. We used immunoprecipitation experiments to test for protein-protein interaction between FAM53A and p53 and did not detect one (data not shown), suggesting an indirect regulatory link between FAM53A and p53. This relationship will require further exploration. It is important to note that in addition to their p53 status, MCF-7 and MDA-MB-231 cells differ in the expression of the ER, K-Ras, and other important cancer signaling proteins, and whether these factors affect the biological behavior of FAM53A in breast cancer remains to be determined.

Our understanding of the role of FAM53A in cancer tumorigenesis and progression is limited. Taken together, our study demonstrates for the first time that FAM53A affects the proliferation, migration, and invasion of breast cancer cells in a p53-dependent manner. These findings suggest that FAM53A has broad prospects in cancer research, particularly in P53 wild-type and P53 mutant breast cancer.

## Data Availability Statement

The raw data supporting the conclusions of this manuscript will be made available by the authors, without undue reservation, to any qualified researcher.

## Ethics Statement

Patients whose tissue samples were used in this research provided written informed consent. This study was approved by the local institutional review board of China Medical University.

## Author Contributions

XM, MS, MH, KD, and JZ contributed conception and design of the study. JZ, MS, MH, JW, SL, and QC performed experiment and the statistical analysis. JZ and MS wrote the first draft of the manuscript. All authors contributed to manuscript revision, read, and approved the submitted version.

### Conflict of Interest

The authors declare that the research was conducted in the absence of any commercial or financial relationships that could be construed as a potential conflict of interest.

## Supplementary Material

Table S1 Antibodies used for western blotting.

Figure S1 FAM53A does not regulate MCF-7 and MDA-MB-231 cells via the Wnt/β-catenin pathway. **(A)** In MCF-7 cells, up- or down-regulation of FAM53A levels did not significantly change β-catenin and glycogen synthase kinase 3β levels. **(B)** In MDA-MB-231 cells, changes in Wnt/β-catenin pathway-related proteins did not show any clear trends.

Figure S2 FAM53A has the opposite effect on A549 and SK-BR3. In the p53-wild-type lung cancer cell line A549(ABC), FAM53A inhibits the ERK pathway and inhibits its proliferation, migration and invasion. While in the p53-mutant breast cancer cell line SK-BR3(ABC), FAM53A promotes the ERK pathway and promotes its proliferation migration and invasion.
