# Supplementary material for: FAM53A Affects Breast Cancer Cell Proliferation, Migration, and Invasion in a p53-Dependent Manner
Source: Front Oncol. 2019 Nov 14;9:1244. doi: 10.3389/fonc.2019.01244 (PMC6874147; doi:10.3389/fonc.2019.01244)
Supplement: Table S1 — Antibodies used for western blotting. [file Table_1.docx]

Supplementary Material

# Supplementary Table

Table S1. Antibodies used for western blotting

| Antibody name | Source | Catalog number | Host | Dilution |
| --- | --- | --- | --- | --- |
| FAM53A | Atlas Antibodies | HPA036452 | Rabbit | 1:1000 |
| GAPDH | Beyotime | AF0006 | Mouse | 1:1000 |
| RhoA | Cell Signaling Technology | 2117 | Rabbit | 1:500 |
| RhoB | Cell Signaling Technology | 2098 | Rabbit | 1:500 |
| RhoC | Cell Signaling Technology | 3430 | Rabbit | 1:500 |
| ROCK1 | Wanlei Bio. | Wl01761 | Rabbit | 1:500 |
| MMP9 | Cell Signaling Technology | 13667 | Rabbit | 1:500 |
| Cyclin D1 | Cell Signaling Technology | 2978 | Rabbit | 1:500 |
| CDK4 | Cell Signaling Technology | 12790 | Rabbit | 1:500 |
| p21 | Proteintech | 10,355-I-AP | Rabbit | 1:1000 |
| c-Myc | Cell Signaling Technology | 13987 | Rabbit | 1:500 |
| ZO-1 | Cell Signaling Technology | 8193 | Rabbit | 1:500 |
| TCF8/ZEB1 | Cell Signaling Technology | 3396 | Rabbit | 1:500 |
| E-Cadherin | Cell Signaling Technology | 3195 | Rabbit | 1:500 |
| N-Cadherin | Cell Signaling Technology | 13116 | Rabbit | 1:500 |
| Vimentin | Cell Signaling Technology | 5741 | Rabbit | 1:1000 |
| ERK1/2 | Cell Signaling Technology | 4695 | Rabbit | 1:500 |
| Phospho-ERK1/2 (Thr202/Tyr204) | Cell Signaling Technology | 4370 | Rabbit | 1:500 |
| Pan-Ras | Proteintech | 60309-1-lg | Mouse | 1:500 |
| K-Ras | Proteintech | 12063-1-AP | Rabbit | 1:500 |
| c-Raf | Cell Signaling Technology | 9422 | Rabbit | 1:500 |
| EGFR | Cell Signaling Technology | 4267 | Rabbit | 1:1000 |
| Phospho-EGFR (Tyr1068) | Cell Signaling Technology | 3777 | Rabbit | 1:500 |
| MEK1/2 | Cell Signaling Technology | 4694 | Mouse | 1:500 |
| Phospho-MEK1/2  (Ser217/221) | Cell Signaling Technology | 3958 | Rabbit | 1:500 |
| p53 | Proteintech | 10,442-I-AP | Rabbit | 1:500 |
