# Supplementary figures and images for: FAM53A Affects Breast Cancer Cell Proliferation, Migration, and Invasion in a p53-Dependent Manner
Source: Front Oncol. 2019 Nov 14;9:1244. doi: 10.3389/fonc.2019.01244 (PMC6874147; doi:10.3389/fonc.2019.01244)

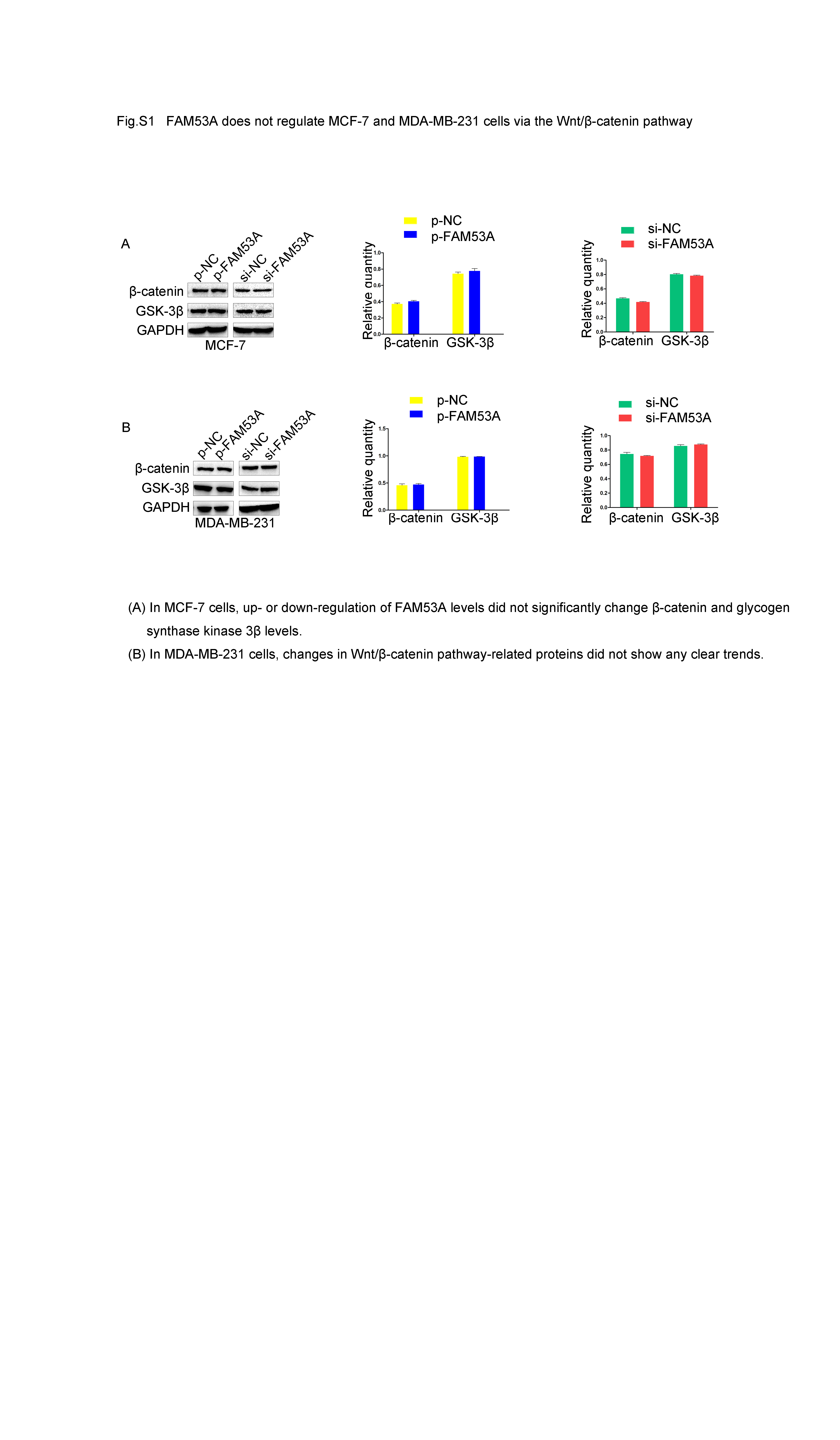

Supplement: Figure S1 — FAM53A does not regulate MCF-7 and MDA-MB-231 cells via the Wnt/β-catenin pathway. (A) In MCF-7 cells, up- or down-regulation of FAM53A levels did not significantly change β-catenin and glycogen synthase kinase 3β levels. (B) In MDA-MB-231 cells, changes in Wnt/β-catenin pathway-related proteins did not show any clear trends. [file Image_1.JPEG]

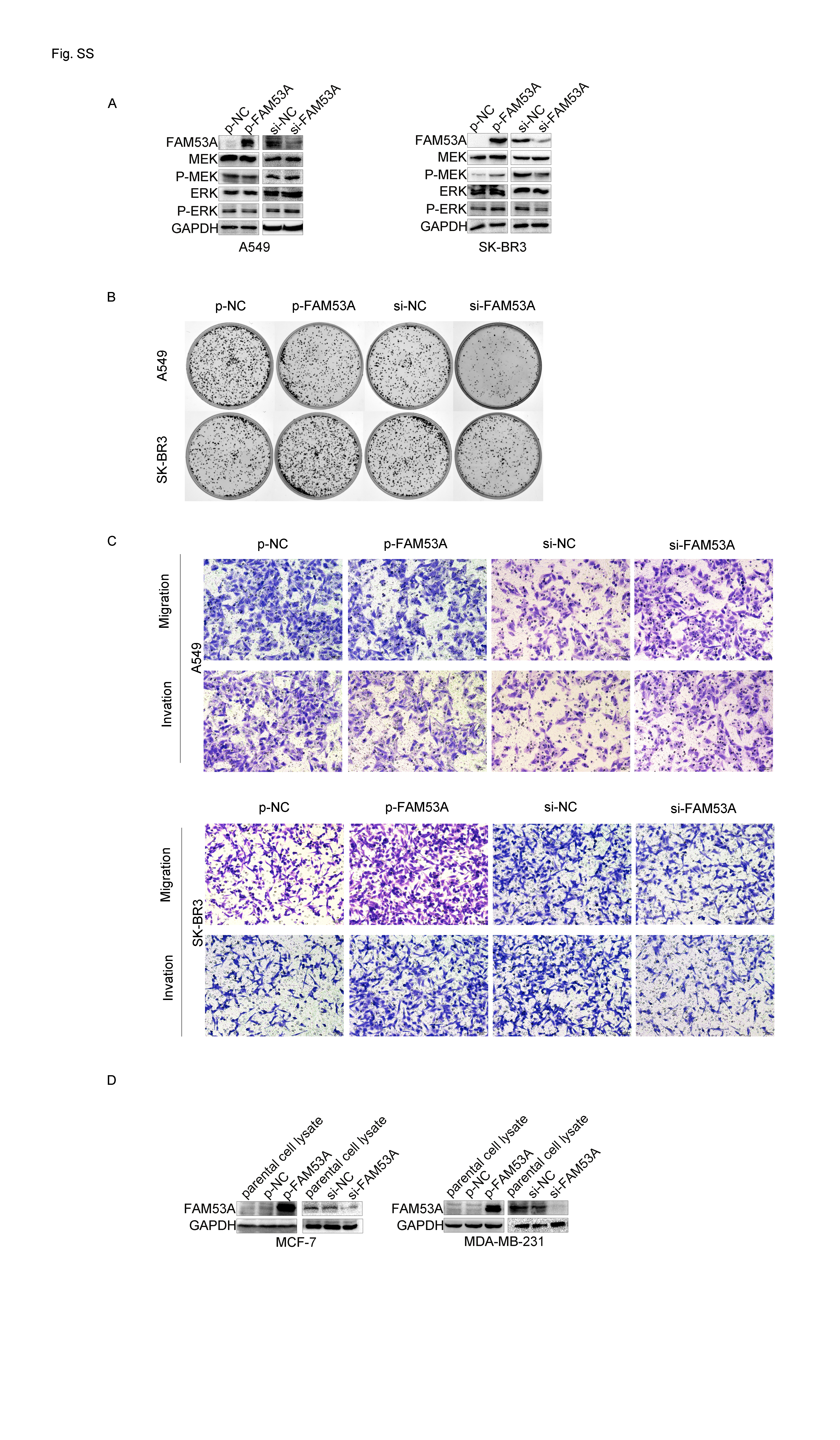

Supplement: Figure S2 — FAM53A has the opposite effect on A549 and SK-BR3. In the p53-wild-type lung cancer cell line A549(ABC), FAM53A inhibits the ERK pathway and inhibits its proliferation, migration and invasion. While in the p53-mutant breast cancer cell line SK-BR3(ABC), FAM53A promotes the ERK pathway and promotes its proliferation migration and invasion. [file Image_2.JPEG]
